# Effects of Naringenin on Diabetic Nephropathy: A Systematic Review and Meta‐Analysis of Rodent Studies

**DOI:** 10.1002/fsn3.72278

**Published:** 2026-08-24

**Authors:** Yuzhu Qu, Jie Chen, Ling Yue, Xinyue Zhang, Kongmei Ou, Yiting Wang, Runjia He, Benxiang He

**Affiliations:** ^1^ Post‐Doctoral Scientific Research Workstation of Affiliated Sport Hospital Affiliated Sport Hospital of Chengdu Sport University Chengdu Sichuan China; ^2^ Acupuncture and Tuina School Chengdu University of Traditional Chinese Medicine Chengdu Sichuan China; ^3^ Hemodialysis Center Pidu District Hospital of Traditional Chinese Medicine Chengdu Sichuan China; ^4^ Department of Rehabilitation Medicine Chengdu Bayi Orthopedic Hospital Chengdu Sichuan China; ^5^ Key Laboratory of Acupuncture for Senile Disease (Chengdu University of TCM), Ministry of Education Chengdu Sichuan China; ^6^ Sichuan Academy of Chinese Medicine Sciences Chengdu Sichuan China

**Keywords:** diabetic nephropathy, meta‐analysis, naringenin, renal protection, systematic review

## Abstract

Diabetic nephropathy (DN) is a common complication of diabetes. Numerous preclinical studies have yielded inconsistent results in the treatment of DN with NAR. This review aimed to evaluate the effects of NAR on rodent models of DN, providing insights for future research and clinical translation. International biomedical and authoritative Chinese databases were searched to identify relevant records from their inception to 30 November 2025. Random‐effects meta‐analysis was performed to evaluate the standard mean difference (SMD) and 95% confidence interval (CI), with *p* ≤ 0.05 indicating statistical significance. Subgroup and sensitivity analyses were performed to assess the robustness of the results. Twelve studies were included in this meta‐analysis. Pooled analysis mainly indicated that NAR reduced blood glucose (SMD = −3.10, 95% CI [−4.48, −1.73]), and protected the renal function (24‐h urinary protein: SMD = −4.86, 95% CI [−5.85, −3.87]; serum creatinine: SMD = −4.51, 95% CI [−6.60, −2.41]). NAR also alleviated renal oxidative stress (malondialdehyde: SMD = −2.84, 95% CI [−4.62, −1.07]; glutathione: SMD = 1.73, 95% CI [0.62, 2.84]) and fibrosis (fibronectin: SMD = −6.60, 95% CI [−12.07, −1.12]; transforming growth factor‐β1: SMD = −4.42, 95% CI [−8.05, −0.78]). These findings suggest that NAR may produce potential multi‐target renal protective effects in DN by regulating blood glucose, inhibiting renal oxidative stress and fibrosis. This study was registered at PROSPERO as CRD420251274617.

## Graphical abstract

Naringenin produces comprehensive renoprotective effects in rodent models of diabetic nephropathy. It mainly lowers blood sugar and alleviates renal dysfunction by reducing proteinuria, serum creatinine, and blood urea nitrogen. Meanwhile, naringenin improves antioxidant status by elevating glutathione and reducing malondialdehyde, and suppresses fibrosis by downregulating fibronectin and transforming growth factor‐β1.

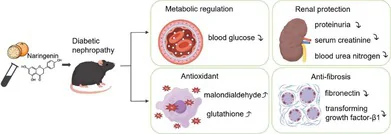

## 1 Introduction

The increasing prevalence of diabetic nephropathy (DN) globally is becoming an increasingly severe public health challenge. DN is the most common microvascular complication of diabetes, with approximately 12%–55% of cases progressing to end‐stage renal disease (ESRD) (Kulkarni and Suryavanshi 2021), and DN is also one of the leading causes of death from ESRD worldwide (Alicic et al. 2017). The “KDIGO 2024 Clinical Practice Guidelines for the Evaluation and Management of Chronic Kidney Disease” emphasize that the prevention and treatment of DN should focus on cardiovascular and renal risk factors, as well as the management of blood glucose, lipids, and blood pressure (Kidney Disease: Improving Global Outcomes (KDIGO) CKD Work Group. 2024). Currently, clinical treatment for DN primarily involves synthetic medications, including renin‐angiotensin‐aldosterone system inhibitors, sodium‐glucose cotransporter‐2 inhibitors, glucagon‐like peptide‐1 receptor agonists, and mineralocorticoid receptor antagonists. However, these drugs have limitations, such as limited efficacy, numerous unpleasant effects, and poor patient adherence, which still cant not meet clinical needs (Bandak et al. 2017; Tonneijck et al. 2016; Xu et al. 2022). Therefore, the pursuit of safe and effective new therapeutic strategies has become a critical area of research.

Naringenin (NAR) is a natural polyphenolic flavonoid compound widely found in citrus fruits, certain plants, and traditional medicinal herbs in two primary forms: aglycone (i.e., naringenin) and glycoside (principally naringin) (Valle‐Velázquez et al. 2024). NAR (naringenin & naringin) possesses a broad range of pharmacological potentials, including regulation of glucose and lipid metabolism, anti‐inflammatory, antioxidant, antifibrotic, immune‐modulatory, anticancer, and neuroprotective effects (Shin and Shin 2024; Xu et al. 2023; Zeng et al. 2018). Their significant pharmacological values have made it a promising therapeutic agent for various diseases, such as diabetes, fatty liver, and breast cancer. Increasing preclinical evidence suggests that NAR can exert protective effects in diabetic kidney injury by regulating glucose and lipid metabolism and oxidative stress. While some studies have reported that NAR does not show significant therapeutic effects in DN. For instance, certain studies found that treatment significantly reduced body weight in DN rats (Yang et al. 2018), whereas another study indicated an increase in body weight after the treatment in DN rats (Kulkarni and Suryavanshi 2021). Some research investigated that NAR could elevate SOD levels in the kidney tissue of DN rats (Roy et al. 2016); conversely, another study reported a reduction in SOD in the kidney tissue of DN rats following the treatment (Pérez et al. 2023). Given the inconsistent results regarding the protective effects of NAR on DN reported in experimental studies, it is crucial to conduct a comprehensive and systematic evaluation of the existing literature to clarify the impact of these compounds on DN.

As an objective analytical method based on statistical principles, meta‐analysis has a profound impact across various research fields. Meta‐analysis allows for the quantitative and scientific synthesis of results from multiple studies with the same research objectives, resolving seemingly contradictory findings and drawing comprehensive conclusions (Gurevitch et al. 2018). Therefore, this study performed a systematic review and meta‐analysis to comprehensively and objectively assess the impact of NAR on DN in rodent models. It aimed to evaluate the renal protective effects of these compounds on DN, providing valuable information for future preclinical and clinical research.

## 2 Methods and Materials

### 2.1 Search Strategy

A comprehensive literature search was conducted across major academic databases, including international biomedical databases (PubMed, Web of Science, Embase, Cochrane Library) and authoritative Chinese medical platforms (CNKI, Wanfang, VIP). The search timeframe covered from the inception of the databases until November 30, 2025. Search terms included “naringenin,” “naringin,” “Diabetic Nephropathy,” “Diabetic Kidney Disease,” etc. Both MeSH and free‐text terms were used for the search, and potential relevant articles or gray literature that may have been missed in the included studies' reference lists were also searched. An example of the search strategy using PubMed is provided in Table 1.

**TABLE 1:** PubMed search strategy.

Search	PubMed
#1	(Diabetic Nephropathies[MeSH Terms]) OR ((((((((((((((((((Diabetic Nephropathies) OR (Nephropathies, Diabetic)) OR (Nephropathy, Diabetic)) OR (Diabetic Kidney Disease)) OR (Diabetic Kidney Diseases)) OR (Kidney Disease, Diabetic)) OR (Kidney Diseases, Diabetic)) OR (Diabetic Nephropathy)) OR (Diabetic Glomerulosclerosis)) OR (Glomerulosclerosis, Diabetic)) OR (Intracapillary Glomerulosclerosis)) OR (Kimmelstiel‐Wilson Disease)) OR (Kimmelstiel Wilson Disease)) OR (Nodular Glomerulosclerosis)) OR (Glomerulosclerosis, Nodular)) OR (Kimmelstiel‐Wilson Syndrome)) OR (Kimmelstiel Wilson Syndrome)) OR (Syndrome, Kimmelstiel‐Wilson))
#2	((naringenin [Supplementary Concept]) OR (naringin [Supplementary Concept])) OR ((Naringenin) OR (Naringin))
#3	#1 AND #2

### 2.2 Selection Criteria

The inclusion criteria were established based on the PICOS principle: (1) P (Population): DN. (2) I (Intervention): naringenin or naringnin treatment, regardless of administration route, dosage, or treatment duration, including both standalone therapy or combination with other treatments. (3) C (Comparison): The control group should either receive no treatment or be administered a placebo/excipient. (4) O (Outcome): The study should include at least one of the renal protection indicators, such as blood glucose, body weight, kidney index, 24‐h urinary protein, urinary albumin, creatinine, urea, albumin, etc. (5) S (Study Design): Only preclinical experimental studies using rodent models of DN will be included, with no language restrictions.

Exclusion criteria: (1) Duplicate publications or studies with overlapping data. (2) Nonanimal experimental designs, including clinical studies and in vitro experiments. (3) Experimental designs that are not controlled or use crossover designs. (4) Studies where the full text or research data cannot be obtained. (5) Studies investigated other NAR derivatives (e.g., methylated or sulfated metabolites) or combinations where naringenin & naringnin cannot be distinguished from other active compounds.

### 2.3 Data Extraction

The literature collection and organization were independently performed by two researchers (Ling Yue and Oukong Mei). Endnote 21 software was used to manage the retrieved references and remove duplicates. Initially, titles and abstracts were reviewed to exclude studies that clearly did not meet the inclusion criteria. The full texts of the remaining studies were then carefully read to select those that met the criteria. The two researchers independently extracted data from the included studies, including the article title, author names, publication date, animal model, sample size, intervention measures, outcome indicators, and other relevant details. According to the Cochrane Handbook for Systematic Reviews of Interventions (CHSRI), if multiple dosage groups were implemented in a study, data from the highest dose group were prioritized for extraction. Longitudinal outcome data were collected from endpoint measurements, and the sample size for the control group was proportionally allocated to avoid data inflation in multigroup designs. Once the data extraction was completed, the results were cross‐checked, and any discrepancies were resolved through consultation with a third reviewer (Xinyue Zhang).

### 2.4 Quality Assessment

Two researchers (Yiting Wang and Runjia He) independently conducted a quality assessment of the included studies using the risk of bias tool developed by the Systematic Review Center for Laboratory Animal Experimentation (SYRCLE). The tool consists of 10 items: (a) sequence generation; (b) baseline characteristics; (c) allocation concealment; (d) random housing; (e) blinding of caregivers; (f) random outcome assessment; (g) outcome assessor blinding; (h) data reporting completeness; (i) selective outcome reporting; (j) other sources of bias. Each item was categorized as “low risk of bias” (“yes”), “high risk of bias” (“no”), or “unclear risk of bias” (“unclear”). In the case of discrepancies in the evaluation results, both researchers would jointly review the original data to reach a consensus. If consensus could not be achieved, the issue would be resolved through consultation with a third researcher (Xinyue Zhang).

### 2.5 Data Synthesis and Analysis

Meta‐analysis was conducted using Review Manager 5.4, with an assessment of heterogeneity and sensitivity analysis between studies. For continuous data, the standard mean difference (SMD) was used as the statistical measure. Given the variations in animal species, intervention methods, dosages, intervention durations, and measurement techniques, a random‐effects model was applied to analyze the combined effect sizes and their 95% confidence intervals (CI). Heterogeneity among the studies was evaluated, with *I* ^2^ ≤ 50% and *p* ≥ 0.1 indicating no significant heterogeneity, and *I* ^2^ > 50% or *p* < 0.1 indicating substantial heterogeneity. Sensitivity analysis or subgroup analysis was performed using the leave‐one‐out method to identify the sources of heterogeneity. When more than 10 studies were included in a subgroup, Egger's plot and the trim‐and‐fill method were used to assess potential publication bias (Jennions and Møller 2002).

## 3 Results

### 3.1 Study Selection

**FIGURE 1:**
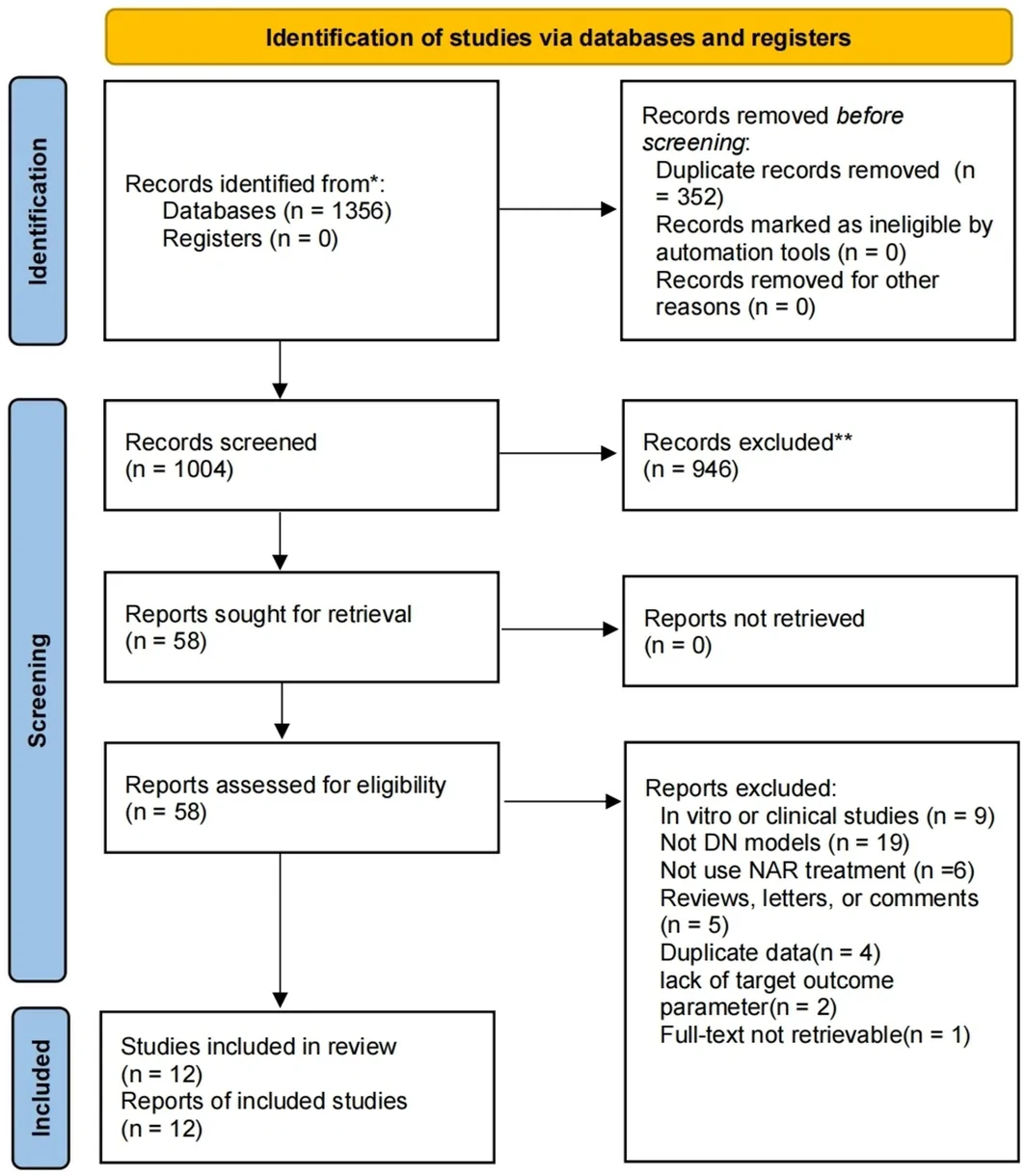
Flowchart for the selection of studies.

Based on PRISMA guidelines, a total of 1356 studies were retrieved (PubMed = 124, Web of Science = 237, Embase = 193, Cochrane Library = 67, CNKI = 393, Wanfang = 121, and VIP = 221). After removing duplicates, 1004 studies remained. Following the title/abstract screening, 58 studies were selected for full‐text review. Ultimately, 12 studies met the inclusion criteria and were included in the meta‐analysis. The screening flow diagram is shown in Figure 1.

### 3.2 Study Characteristics

The 12 studies included in this meta‐analysis were published between 2016 and 2023. The animal models used in these studies comprised various strains of rats and mice, including Wistar rats (three studies), Sprague Dawley rats (four studies), C57BL/6J mice (one study), C57BL/6 mice (one study), db/db mice (two studies), and *Mus musculus castaneus* mice (one study). All models were induced with DN via streptozotocin. The intervention groups received naringenin (nine studies) or naringnin (three studies) treatments through oral gavage, with doses ranging from 5 to 200 mg and treatment durations ranging from 2 weeks to 12 weeks. The control groups were administered an equal volume of distilled water, saline, 0.5% carboxymethyl cellulose via gavage, or solvent injections. There were variations among the studies in terms of dosage, timing, and duration of administration, outcome measures, and primary conclusions. Detailed study characteristics are presented in Table 2.

**TABLE 2:** Characteristics of included studies.

Study	Species (sex, *n* = I/C), weight	Model	Intervention	Control	Duration	Main outcomes	Main conclusion
Roy et al. (2016)	Wistar rats (male, 10/10), 140–180 g	Injection STZ 50 mg/kg	Naringenin, 5 or 10 mg/kg, gavage	Distilled water gavage	Once daily, 10 weeks	①②⑤⑥⑦⑫⑬⑮⑯⑱	NAR protected diabetic renal impairment by altering oxidative stress, modulation of cytokines expression and apoptotic events.
Yan et al. (2016)	SD rats (male,10/8), 120 ± 20 g	Injection STZ 30 mg/kg	Naringenin, 50 mg/kg, gavage	Saline gavage	Once daily, 6 weeks	①③④⑨⑰⑱	NAR ameliorated kidney injury by regulating let‐7a/TGFBR1 signaling
Rajappa et al. (2017)	Swiss albino mice (male, 6/6), 25–30 g	Injection STZ 50 mg/kg	Naringenin, 50 or 100 mg/kg, gavage	0.5% CMC gavage	Once daily, 45 days	⑤⑥⑦⑪⑫⑬⑮⑯	NAR mitigated kidney complications in DN by promoting antioxidant defense enzymes and increasing GSH
Zhang et al. (2017)	SD rat (male, 10/10), 200–250 g	Injection STZ 60 mg/kg	Naringnin, 20, 40 or 80 mg/kg, tail‐vein injection	Sterile saline tail‐vein injection	Once daily, 12 weeks	①③④⑤⑩⑫⑬⑭	NAR ameliorated DN by inhibiting NOX4
Yang et al. (2018)	C57BL/6J mice (male, 10/10), NM	Injection STZ50mg/kg	Naringnin, 100 or 200 mg/kg, gavage	Vehicle gavage	Once daily, 6 day/week, 8 weeks	①②③④⑤⑩⑫⑰	NAR alleviated diabetic renal fibrosis by inhibiting the ERK1/2 and JNK MAPK signaling pathways
Qu and Li (2018)	db/db mice (male, 5/5), NM	Spontaneous Type 2 DN model	Naringenin, 50 mg/kg, gavage	Saline gavage	Once daily, 6 weeks	①②⑧	NAR alleviated kidney injury in DN by inhibiting NO and NOS to resist oxidative stress
Li et al. (2018)	db/db mice (male, 5/5), NM	Spontaneous Type 2 DN model	Naringenin, 50 mg/kg, gavage	Saline gavage	Once daily, 2 weeks	①②④⑫	NAR relieved kidney injury via inhibiting the expression of inflammatory factors through NF‐κB in DN
Ding et al. (2019)	*Mus musculus castaneus* mice (male, 10/10), 15–20 g	Injection STZ40mg/kg	Naringenin, 25 or 75 mg/kg, gavage	0.5% CMC‐Na gavage	Once daily, 4 weeks	①④⑤⑩⑫	NAR produced a therapeutic effect on renal injury in DN, which related to the upregulation of CYP4A, the increase of 20‐HETE and the excitation of PPARs
Ran et al. (2019)	SD rats (male, 5/5), 220 g	Injection STZ60mg/kg	Naringenin, 50 or 100 mg/kg, gavage	Saline gavage	Once daily, 4 weeks	①⑤⑩⑬⑭	NAR delayed the kidney injury in DN by inhabiting the expression of inflammatory cytokines and reducing oxidative stress
Xie et al. (2020)	C57BL/6 mice (male, 8/8), 20 ± 1.5 g	Injection STZ80mg/kg	Naringenin, 50 mg/kg, gavage	Saline gavage	Once daily, 12 weeks	①②④⑤⑫⑰	NAR ameliorated the renal fibrosis in DN related to the downregulation of the RhoA/ROCK signaling pathway
Kulkarni and Suryavanshi (2021)	SD rats (male, 6/6), 180–220 g	Injection STZ55mg/kg	Naringenin, 10, 25 or 50 mg/kg, gavage	Vehicle gavage	Once daily, 4 weeks	①②④⑤⑦⑩⑫⑬⑭⑮⑱	NAR was useful in improving DN
Pérez et al. (2023)	Wistar rats (male, 8/8), 150–200 g	Injection STZ 60 mg/kg	Naringnin, 40 mg/kg, gavage	Vehicle gavage	Once daily, 30 days	①②③⑤⑥⑦⑫⑬⑮⑯	NAR produced nephroprotection is mediated through the counteraction of oxidative stress in the mitochondria of proximal tubules

*Note:* Basic characteristics of included studies: ① blood glucose, ② body weight, ③ kidney index, ④ 24‐h urinary protein, ⑤ serum creatinine, ⑥ serum urea, ⑦ serum albumin, ⑧ urinary albumin, ⑨ creatinine clearance, ⑩ blood urea nitrogen, ⑪ uric acid, ⑫ pathological images of renal tissue, ⑬ superoxide dismutase, ⑭ malondialdehyde, ⑮ glutathione, ⑯ catalase, ⑰ fibronectin, ⑱ transforming growth factor‐β1.

Abbreviations: CMC, carboxymethyl cellulose; NM, not mentioned; SD, Sprague–Dawley rats; STZ, streptozotocin.

### 3.3 Risk of Bias and Quality of Included Studies

The included studies exhibited a risk of bias score ranging from 3 to 4 points. Specifically, six studies were rated with a score of 3, while the remaining seven studies received a score of 4. Among the 12 studies, only Roy et al. (2016) clearly described the use of a random number table to generate the allocation sequence; the other 11 studies did not provide specific details on the random sequence generation method. Although all studies reported baseline data such as body weight, blood glucose, and renal function, and demonstrated balance between groups, none of the studies adequately described allocation concealment measures or random housing procedures. Seven studies used different intervention methods (tail vein injection and gavage) but did not clearly report blinding measures for the researchers, while six studies used the same intervention methods but did not provide sufficient information on the implementation of researcher blinding. Furthermore, whether randomization or blinding was applied in outcome assessments was unclear. All studies fully reported primary outcome data, and the outcomes listed in the methods section were consistently reported in the results, with no selective omission. Seven studies did not report sample size calculations or registration information, and the presence of other biases remains unclear. No other biases were found in the remaining six studies. Specific risk of bias scores can be found in Table 3.

**TABLE 3:** Methodological quality assessment of included studies.

Study	A	B	C	D	E	F	G	H	I	J	Total scores
Roy et al. (2016)	+	+	?	?	−	?	?	+	+	?	4
Yan et al. (2016)	?	+	?	?	−	?	?	+	+	?	3
Rajappa et al. (2017)	?	+	?	?	−	?	?	+	+	?	3
Zhang et al. (2017)	?	+	?	?	−	?	?	+	+	?	3
Yang et al. (2018)	?	+	?	?	−	?	?	+	+	?	3
Qu and Li (2018)	?	+	?	?	−	?	?	+	+	?	3
Li et al. (2018)	?	+	?	?	−	?	?	+	+	?	3
Ding et al. (2019)	?	+	?	?	?	?	?	+	+	+	4
Ran et al. (2019)	?	+	?	?	?	?	?	+	+	+	4
Xie et al. (2020)	?	+	?	?	?	?	?	+	+	+	4
Kulkarni and Suryavanshi (2021)	?	+	?	?	?	?	?	+	+	+	4
Pérez et al. (2023)	?	+	?	?	?	?	?	+	+	+	4

*Note:* (1) Selection bias: A, Sequence generation; B, Baseline characterization; C, Allocation concealment. (2) Performance bias: D, Random housing; E, Blinding of caregivers. (3) Detection bias: F, Random outcome assessment; G, Blinding of outcome assessor. (4) Attrition bias: H, Incomplete outcome data reporting. (5) Reporting bias: I, Selective outcome reporting. (6) Other: J, Other sources of bias.?, unclear; +, low risk; −, high risk.

### 3.4 Metabolic Regulation of NAR

#### 3.4.1 Effects on Blood Glucose

**FIGURE 2:**
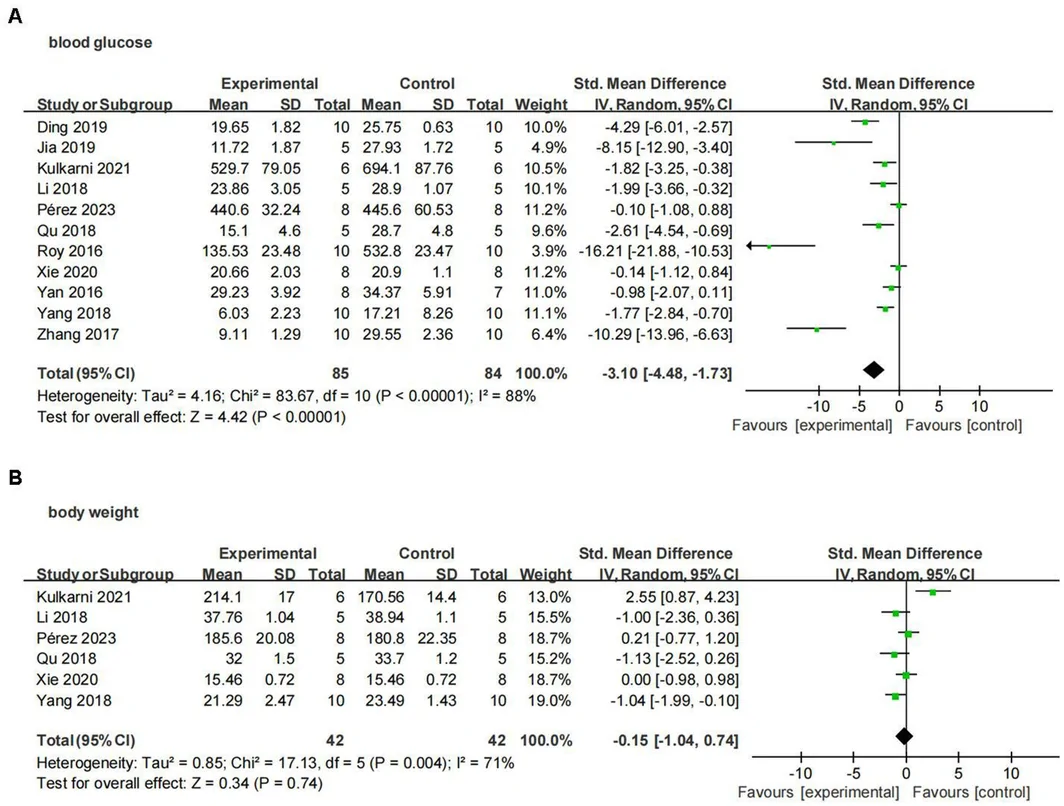
Metabolic regulation of NAR. (A) Effects on blood glucose. (B) Effects on body weight.

A total of 11 studies assessed the effect of NAR on fasting blood glucose levels in DN. The pooled analysis revealed that the NAR group had significantly lower blood glucose levels compared to the control group (SMD = −3.10, 95% CI [−4.48, −1.73], *p* < 0.01). There was substantial heterogeneity between studies (*I* ^2^ = 88%, *p* < 0.01) (Figure 2A).

#### 3.4.2 Effects on Body Weight

A total of six studies investigated the effect of NAR on body weight. The pooled results showed no statistically significant difference in body weight changes between the NAR and control groups (SMD = −0.15, 95% CI [−1.04, −0.74], *p* = 0.74). Heterogeneity analysis revealed significant heterogeneity between the studies (*I* ^2^ = 17.13%, *p* < 0.01) (Figure 2B).

### 3.5 Protection on Renal Function of NAR

#### 3.5.1 Effects on 24 h‐Upro

**FIGURE 3:**
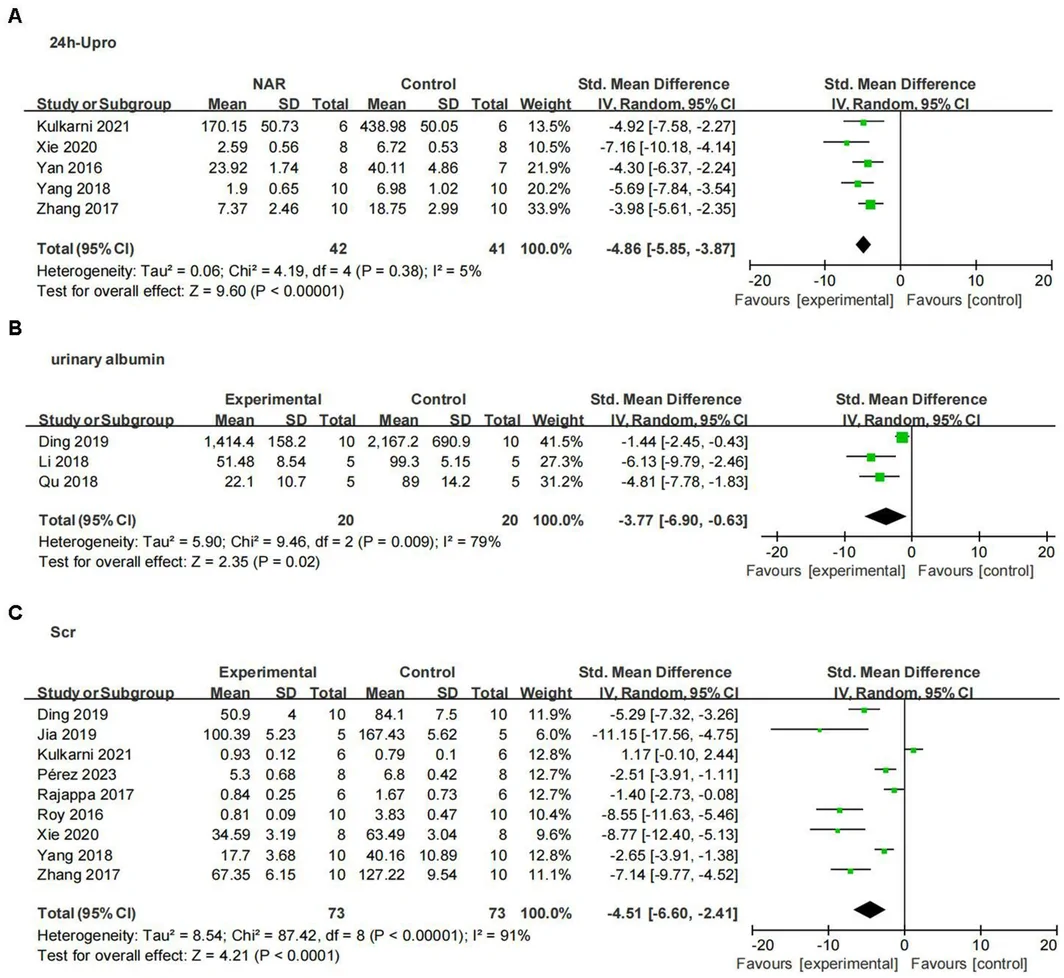
Protection on renal function of NAR. (A) Effects on 24 h‐Upro. (B) Effects on urinary albumin. (C) Effects on Scr. 24 h‐Upro, 24‐h urinary protein; Scr, serum creatinine.

A total of five studies evaluated the effect of NAR on 24‐h urinary protein (24 h‐Upro) levels. The pooled results showed that the NAR group had significantly lower levels of 24 h‐Upro compared to the control group (SMD = −4.86, 95% CI [−5.85, −3.87], *p* < 0.01). Heterogeneity analysis indicated low heterogeneity between the studies (*I* ^2^ = 5%, *p* = 0.38) (Figure 3A).

#### 3.5.2 Effects on Urinary Albumin

A total of 3 studies assessed the effect of NAR on urinary albumin levels. The pooled results showed that the NAR group had significantly lower urinary albumin levels compared to the control group (SMD = −3.77, 95% CI [−6.90, −0.63], *p* < 0.05). Heterogeneity analysis revealed significant heterogeneity between the studies (*I* ^2^ = 79%, *p* < 0.01; Figure 3B).

#### 3.5.3 Effects on Scr

A total of 9 studies evaluated the effect of NAR on serum creatinine (Scr) levels. The pooled analysis revealed that the NAR group had significantly lower Scr levels compared to the control group (SMD = −4.51, 95% CI [−6.60, −2.41], *p* < 0.01). There was significant heterogeneity between the studies (*I* ^2^ = 91%, *p* < 0.01; Figure 3C).

#### 3.5.4 Effects on Serum Urea

**FIGURE 4:**
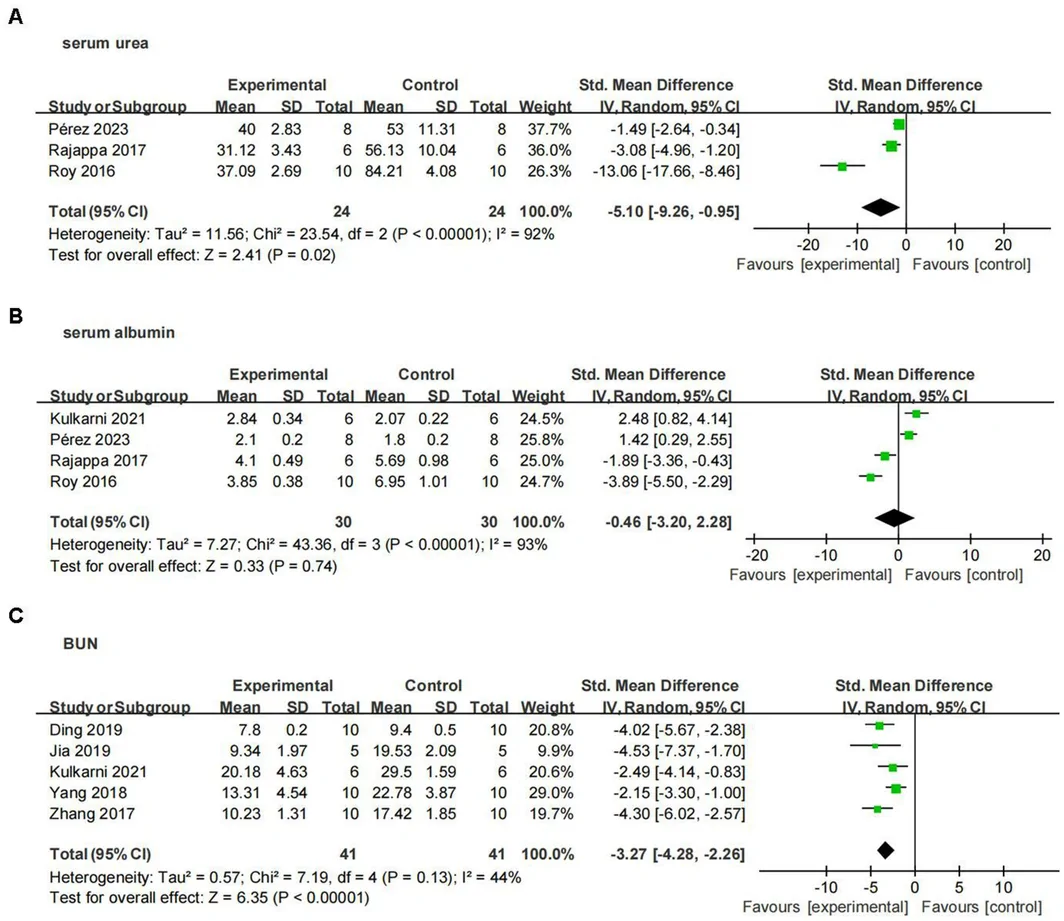
Protection on renal function of NAR. (A) Effects on serum urea. (B) Effects on serum albumin. (C) Effects on BUN. BUN, blood urea nitrogen.

A total of 3 studies assessed the effect of NAR on serum urea levels. The pooled results showed that the NAR group had significantly lower serum urea levels compared to the control group (SMD = −5.10, 95% CI [−9.26, −0.95], *p* < 0.05). Heterogeneity analysis revealed significant heterogeneity between the studies (*I* ^2^ = 92%, *p* < 0.01; Figure 4A).

#### 3.5.5 Effects on Serum Albumin

A total of 4 studies evaluated the effect of NAR on serum albumin levels. The pooled results showed no significant difference in serum albumin levels between the NAR and control groups (SMD = −0.46, 95% CI [−3.20, 2.28], *p* = 0.74). Heterogeneity analysis revealed significant heterogeneity between the studies (*I* ^2^ = 93%, *p* < 0.01; Figure 4B).

#### 3.5.6 Effects on BUN

A total of 5 studies assessed the effect of NAR on blood urea nitrogen (BUN) levels. The pooled analysis revealed that the NAR group had significantly lower BUN levels compared to the control group (SMD = −3.27, 95% CI [−4.28, −2.26], *p* < 0.01). Heterogeneity analysis indicated moderate heterogeneity between the studies (*I* ^2^ = 44%, *p* = 0.13; Figure 4C).

### 3.6 Antioxidant Effects of NAR

#### 3.6.1 Effects on SOD

**FIGURE 5:**
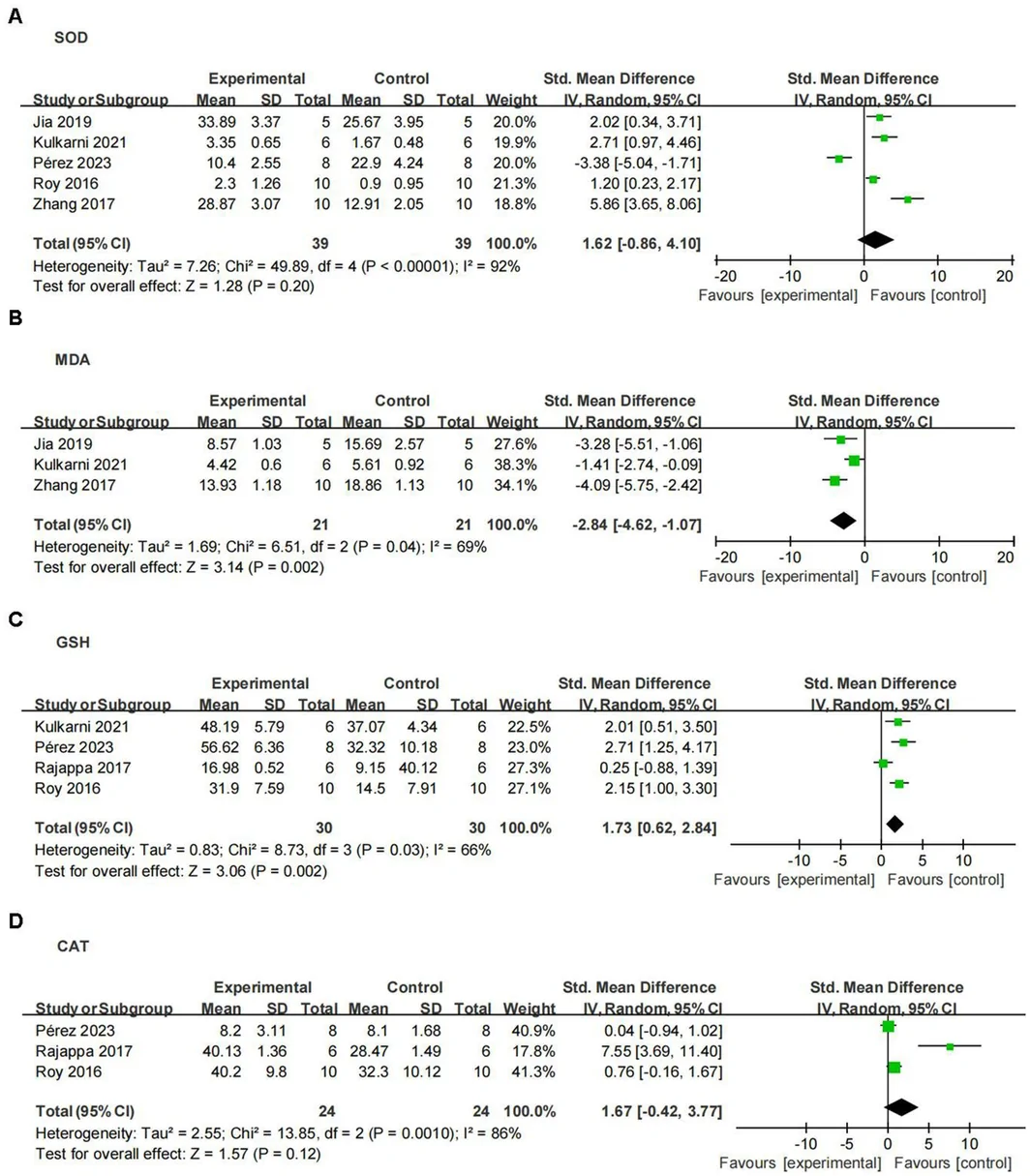
Antioxidant effect of NAR. (A) Effects on SOD. (B) Effects on MDA. (C) Effects on GSH. (D) Effects on CAT. CAT, catalase; GSH, glutathione; MDA, malondialdehyde; SOD, superoxide dismutase.

A total of five studies investigated the effect of NAR on SOD levels in renal tissue. The pooled results showed no statistically significant difference in SOD changes between the NAR and control groups (SMD = 1.62, 95% CI [−0.86, 4.10], *p* = 0.20). Heterogeneity analysis revealed significant heterogeneity between the studies (*I* ^2^ = 17.13%, *p* < 0.01; Figure 5A).

#### 3.6.2 Effects on MDA

A total of three studies assessed the effect of NAR on malondialdehyde (MDA) levels in renal tissue. The pooled results showed that the NAR group had significantly lower MDA levels compared to the control group (SMD = −2.84, 95% CI [−4.62, −1.07], *p* < 0.01). Heterogeneity analysis revealed significant heterogeneity between the studies (*I* ^2^ = 69%, *p* < 0.05; Figure 5B).

#### 3.6.3 Effects on GSH

A total of 4 studies assessed the effect of NAR on glutathione (GSH) levels in renal tissue. The pooled results showed that the NAR group had significantly higher GSH levels compared to the control group (SMD = 1.73, 95% CI [0.62, 2.84], *p* < 0.01). Heterogeneity analysis revealed significant heterogeneity between the studies (*I* ^2^ = 66%, *p* < 0.05; Figure 5C).

#### 3.6.4 Effects on CAT

A total of three studies assessed the effect of NAR on CAT levels in renal tissue. The pooled results showed no statistically significant difference in CAT changes between the NAR and control groups (SMD = 1.67, 95% CI [−0.42, 3.77], *p* = 0.12). Heterogeneity analysis revealed significant heterogeneity between the studies (*I* ^2^ = 86%, *p* < 0.01; Figure 5D).

### 3.7 Anti‐Fibrosis in the Kidney of NAR

#### 3.7.1 Effects on FN

**FIGURE 6:**
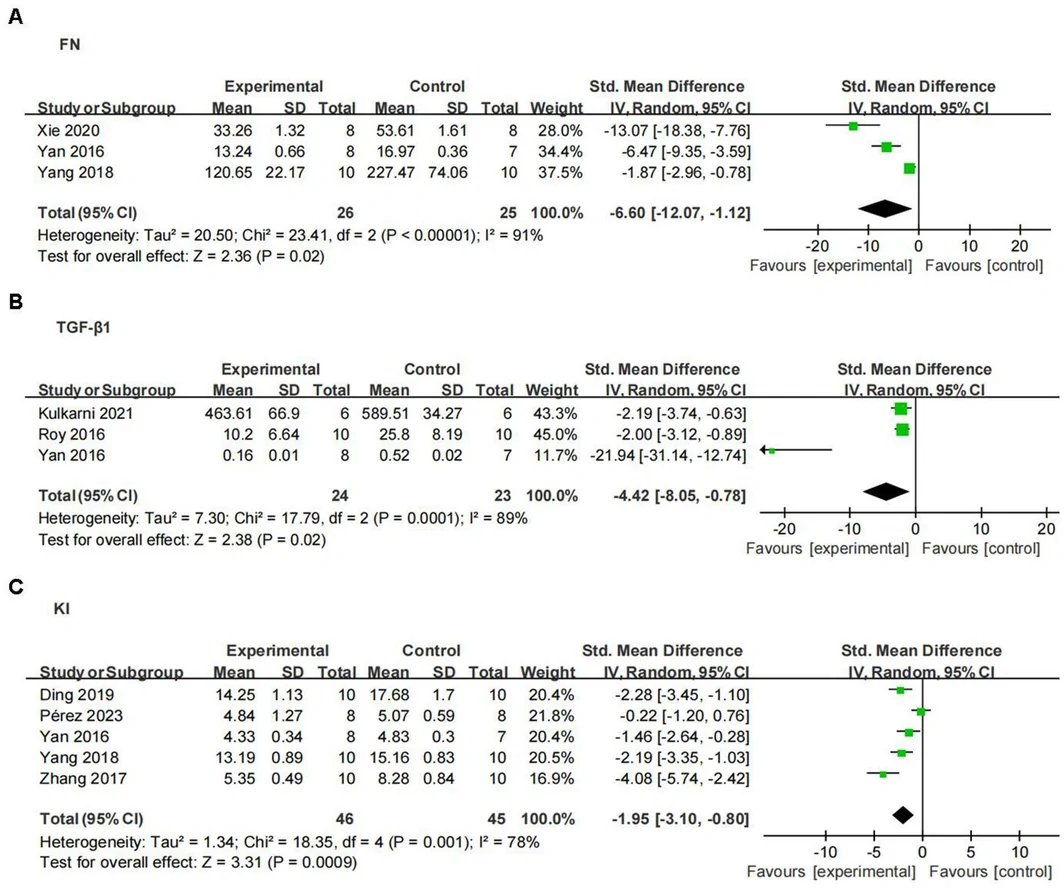
Anti‐fibrosis in the kidney of NAR. (A) Effects on FN. (B) Effects on TGF‐β1. (C) Effects on KI. FN, fibronectin; KI, kidney index; TGF‐β1, transforming growth factor‐β1.

A total of three studies assessed the effect of NAR on fibronectin (FN) levels in renal tissue. The pooled results showed that the NAR group had significantly lower FN levels compared to the control group (SMD = −6.60, 95% CI [−12.07, −1.12], *p* < 0.05). Heterogeneity analysis revealed significant heterogeneity between the studies (*I* ^2^ = 91%, *p* < 0.01; Figure 6A).

#### 3.7.2 Effects on TGF‐β1

A total of three studies assessed the effect of NAR on transforming growth factor‐β1 (TGF‐β1) levels. The pooled results showed that the NAR group had significantly lower TGF‐β1 levels compared to the control group (SMD = −4.42, 95% CI [−8.05, −0.78], *p* < 0.05). Heterogeneity analysis revealed significant heterogeneity between the studies (*I* ^2^ = 89%, *p* < 0.01; Figure 6B).

#### 3.7.3 Effects on KI

A total of five studies assessed the effect of NAR on kidney index (KI). The pooled analysis showed that the NAR group had significantly lower KI compared to the control group (SMD = −1.95, 95% CI [−3.10, −0.80], *p* < 0.01). Heterogeneity analysis revealed significant heterogeneity between the studies (*I* ^2^ = 78%, *p* < 0.01; Figure 6C).

### 3.8 Subgroup Analysis

Based on the different doses of NAR administered, the daily intervention doses were categorized into low dose (< 50 mg/kg) and high dose (50 mg/kg ≤ dose ≤ 100 mg/kg). Additionally, based on the treatment duration, the studies were divided into short‐term intervention (4 weeks ≤ duration < 8 weeks) and long‐term intervention (8 weeks ≤ duration ≤ 12 weeks). Subgroup analyses were conducted based on both NAR treatment dose and treatment duration. Overall, the results indicated that NAR treatment was beneficial across all subgroups (Table 4).

**TABLE 4:** Subgroup analysis.

Comparison	Subgroup	Number	SMD [95% CI]	*p* (meta‐analysis)	*I* ^2^	*p* (heterogeneity)
Blood glucose						
Dose	Low	5	−4.81 [−7.82, −1.80]	0.002	93%	< 0.001
	High	9	−2.76 [−4.06, −1.46]	< 0.001	84%	< 0.001
Duration	Short	6	−1.69 [−3.28, −0.11]	0.04	86%	< 0.001
	Long	5	−4.95 [−7.92, −1.97]	0.001	93%	< 0.001
Body weight						
Dose	Low	2	1.11 [−0.83, 3.06]	0.26	78%	0.03
	High	5	−0.21 [−1.30, 0.88]	0.71	75%	0.003
Duration	Short	3	0.36 [−1.66, 2.37]	0.73	84%	0.002
	Long	3	−0.67 [−1.41, 0.07]	0.08	29%	0.25
24 h‐Upro						
Dose	Low	2	−2.72 [−4.25, −1.19]	< 0.001	42%	0.19
	High	5	−4.68 [−5.77, −3.59]	< 0.001	25%	0.26
Duration	Short	2	−3.98 [−5.45, −2.51]	< 0.001	0%	0.66
	Long	3	−5.28 [−7.04, −3.53]	< 0.001	48%	0.15
Scr						
Dose	Low	5	−3.62 [−6.02, −1.22]	0.003	91%	< 0.001
	High	7	−4.34 [−6.86, −1.83]	< 0.001	92%	< 0.001
Duration	Short	5	−2.81 [−5.33, −0.29]	0.03	90%	< 0.001
	Long	4	−6.57 [−10.09, −3.05]	< 0.001	88%	< 0.001
Serum albumin						
Dose	Low	3	−0.43 [−3.41, 2.54]	0.78	94%	< 0.001
	High	2	0.28 [−4.01, 4.56]	0.90	93%	< 0.001
Duration	Short	3	0.66 [−1.78, 3.10]	0.60	89%	< 0.001
	Long	/	/	/	/	/
BUN						
Dose	Low	3	−2.59 [−3.98, −1.19]	< 0.001	66%	0.05
	High	5	−2.42 [−4.89, 0.05]	0.05	91%	< 0.001
Duration	Short	3	−1.96 [−6.73, 2.81]	0.42	94%	< 0.001
	Long	2	−3.12 [−5.22, −1.03]	0.003	76%	0.04
SOD						
Dose	Low	4	1.12 [−1.64, 3.87]	0.43	93%	< 0.001
	High	3	3.42 [1.30, 5.54]	0.002	74%	0.02
Duration	Short	3	0.45 [−3.35, 4.25]	0.82	93%	< 0.001
	Long	2	3.42 [−1.14, 7.97]	0.14	93%	< 0.001
GSH						
Dose	Low	3	1.84 [0.74, 2.94]	0.001	56%	0.10
	High	2	1.06 [−0.65, 2.77]	0.22	70%	0.07
Duration	Short	3	1.60 [0.07, 3.13]	0.04	74%	0.02
	Long	/	/	/	/	/
KI						
Dose	Low	3	−1.53 [−3.04, −0.02]	0.05	82%	0.004
	High	4	−2.33 [−3.26, −1.39]	< 0.001	54%	0.09
Duration	Short	3	−1.28 [−2.50, −0.06]	0.04	72%	0.03
	Long	2	−3.04 [−4.88, −1.20]	0.001	70%	0.07

*Note:* Number refers to the number of studies included in the subgroup analysis. Low dose: NAR < 50 mg/kg once daily; High dose: 50 mg/kg ≤ NAR ≤ 100 mg/kg once daily. Short duration: 4 weeks ≤ NAR treatment duration < 8 weeks; Long duration: 8 weeks ≤ NAR treatment ≤ 12 weeks.

Abbreviations: 24 h‐Upro, 24‐h urinary protein; BUN, blood urea nitrogen; GSH, glutathione; KI, kidney index; Scr, serum creatinine; SMD, standardized mean difference; SOD, superoxide dismutase.

#### 3.8.1 Subgroup of Effects on Blood Glucose

Subgroup analysis showed that NAR at a dose of < 50 mg/kg demonstrated better glucose‐lowering effects (*p* = 0.002). Regarding treatment duration, long‐term intervention (8–12 weeks) exhibited more favorable glucose‐lowering effects compared to short‐term intervention (4–8 weeks) (*p* = 0.001).

#### 3.8.2 Subgroup of Effects on Body Weight

Subgroup analysis revealed no statistically significant differences in the effect of NAR on body weight in DN, regardless of whether the dose was high or low, or the intervention was short‐term or long‐term.

#### 3.8.3 Subgroup of Effects on 24 h‐Upro

Subgroup analysis showed that NAR at different doses and treatment durations effectively reduced 24 h‐Upro in DN animals, with higher doses (*p* < 0.001) and longer treatment duration (*p* < 0.001) yielding more significant benefits.

#### 3.8.4 Subgroup of Effects on Scr

Subgroup analysis consistently showed that NAR treatment was beneficial across all subgroups. When analyzing by high and low doses, high‐dose NAR treatment demonstrated the most significant effects (*p* < 0.001). Regarding short‐term versus long‐term treatment durations, long‐term interventions showed more significant benefits (*p* < 0.001).

#### 3.8.5 Subgroup of Effects on Serum Albumin

Due to the insufficient number of studies (less than two) for the 8–12 weeks treatment duration, subgroup analysis for this duration was not performed. However, the remaining subgroup analyses showed that, regardless of whether the dose was high or low, or the intervention was short‐term, NAR treatment failed to significantly improve the serum albumin levels.

#### 3.8.6 Subgroup of Effects on BUN

Subgroup analysis based on intervention dose and treatment duration consistently showed that NAR treatment benefited all subgroups. Compared to high doses, low‐dose NAR treatment yielded more significant benefits (*p* < 0.001). Additionally, long‐term interventions showed more significant effects than short‐term interventions (*p* = 0.003).

#### 3.8.7 Subgroup of Effects on SOD

Subgroup analysis showed that, compared to the low dose, high‐dose NAR treatment had a more significant effect on increasing SOD levels (*p* = 0.002). However, no statistically significant differences were observed between short‐term and long‐term NAR treatments.

#### 3.8.8 Subgroup of Effects on GSH

Due to the insufficient number of studies (less than two) for the 8–12 weeks treatment duration, subgroup analysis for this duration was not performed. However, the remaining subgroup analyses showed that NAR at < 50 mg/kg resulted in more significant improvements in KI (*p* = 0.001). Short‐term intervention (4–8 weeks) also demonstrated significant therapeutic effects (*p* = 0.04).

#### 3.8.9 Subgroup of Effects on KI

Subgroup analysis based on intervention dose and treatment duration showed that NAR treatment benefited all subgroups. High‐dose NAR treatment showed more significant improvements in KI compared to low‐dose treatment (*p* < 0.001). Additionally, long‐term interventions demonstrated more significant therapeutic effects than short‐term interventions (*p* = 0.001).

### 3.9 Sensitivity Analysis and Publication Bias

**FIGURE 7:**
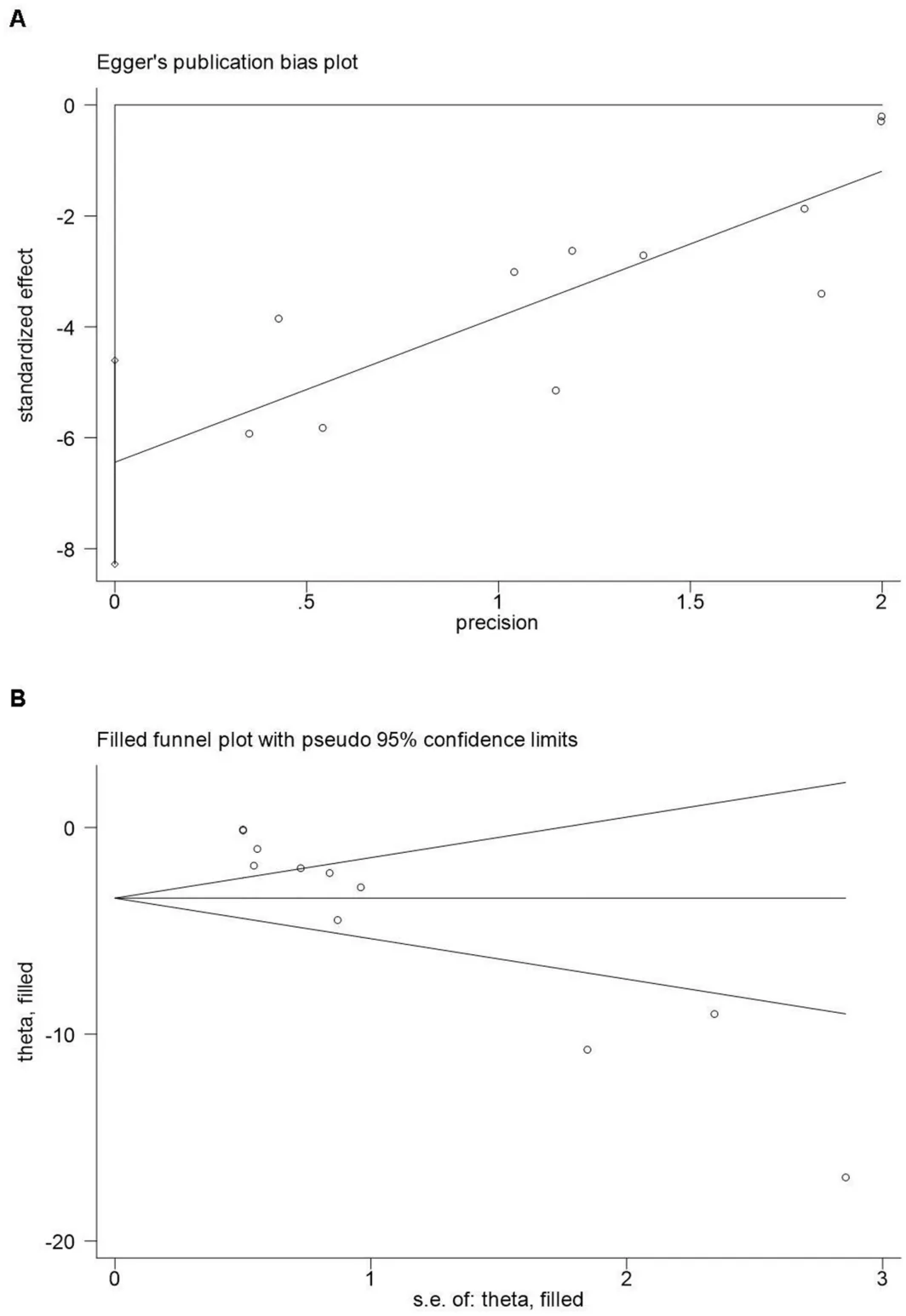
Publication bias. (A) Egger's publication bias of blood glucose. (B) Trim‐and‐fill analysis of the publication bias of blood glucose.

Based on the number of included studies, sensitivity analyses were performed for blood glucose, body weight, 24 h‐Upro, Scr, BUN, SOD, and KI. The leave‐one‐out sensitivity analysis indicated that the pooled effect sizes were robust and not unduly influenced by any single study. Publication bias was assessed for outcomes with more than 10 included studies, specifically blood glucose. The Egger plot demonstrated an asymmetric funnel plot distribution for blood glucose (*p* < 0.001; Figure 7A), suggesting the presence of potential publication bias. Subsequently, the trim‐and‐fill method was applied to evaluate the possible impact of publication bias (Figure 7B). Despite the indication of publication bias, the adjusted effect size was highly consistent with the original pooled estimate, suggesting that this bias is unlikely to materially alter the overall conclusions.

## 4 Discussion

This study is the first to comprehensively evaluate the efficacy and therapeutic potential of NAR (primarily naringenin & naringin) in rodent models of DN through a systematic review and meta‐analysis. The findings from the 12 included studies suggest that NAR may exert protective effects across multiple key stages of DN progression. In terms of metabolic regulation, NAR effectively reduced blood glucose, indicating an improvement in the core pathophysiological features of diabetes. Regarding renal function, NAR significantly decreased Scr, BUN, 24 h‐Upro, and urinary albumin levels, suggesting improvements in glomerular filtration function and attenuation of glomerular barrier damage. From a renal histopathological perspective, NAR alleviated renal hypertrophy by reducing the KI and downregulated the expression of fibrosis‐related markers, including FN and TGF‐β1, indicating a benefit in suppressing renal structural remodeling and fibrosis. With respect to oxidative stress, NAR significantly reduced MDA and increased GSH in renal tissue, confirming its ability to effectively alleviate oxidative stress in DN. All outcome measures exhibited varying degrees of heterogeneity, and subgroup analyses suggested that this heterogeneity may be attributable to differences in animal species, dosing regimens, and treatment duration. Sensitivity analyses indicated that the pooled results were robust and not unduly influenced by any single study. Although the pooled estimates suggest a beneficial signal of NAR in rodent DN models, these findings must be interpreted with substantial caution. The existing evidence base is restricted to preclinical studies with small sample sizes, considerable methodological heterogeneity, and generally low‐to‐moderate reporting quality.

### 4.1 NAR May Mediate a Comprehensive Protective Effect on the Renal

**FIGURE 8:**
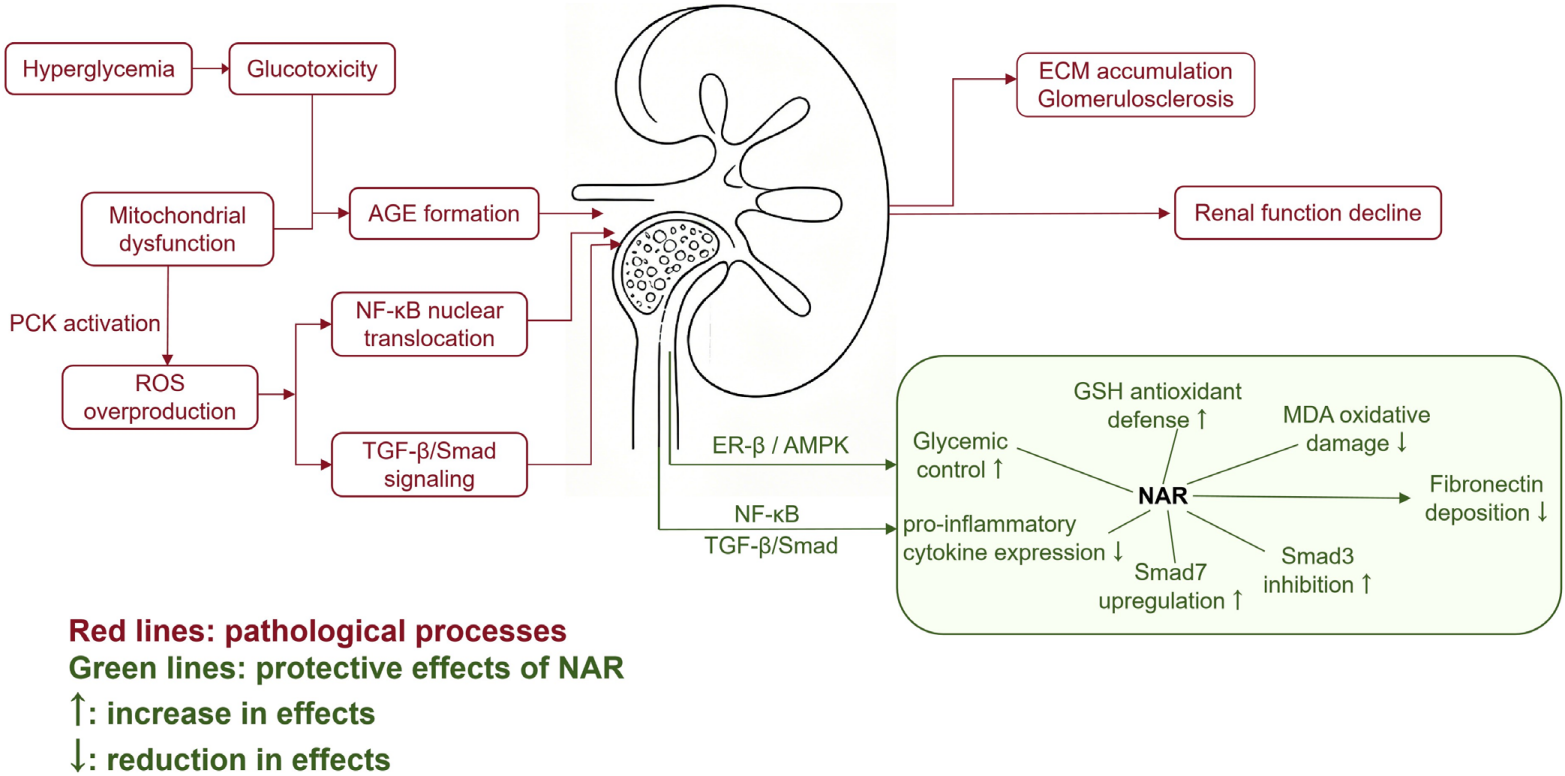
NAR‐mediated comprehensive protective effects on DN. Sustained hyperglycemia initiates glucotoxicity, which promotes mitochondrial dysfunction and reactive oxygen species (ROS) overproduction. ROS activates protein kinase C (PKC), advanced glycation end product (AGE) formation, and the polyol pathway, while simultaneously triggering NF‐κB–dependent inflammatory cascades and TGF‐β/Smad‐mediated fibrotic signaling. These interconnected pathways converge on extracellular matrix (ECM) accumulation, glomerulosclerosis, and progressive renal function decline. NAR intervenes at multiple nodes: (1) it improves glycemic control via estrogen receptor β (ER‐β) and AMP‐activated protein kinase (AMPK) pathways, reducing glucotoxicity; (2) it enhances antioxidant defenses (GSH) and reduces lipid peroxidation (MDA), thereby attenuating ROS‐driven signaling; (3) it inhibits NF‐κB nuclear translocation and downstream pro‐inflammatory cytokine expression; and (4) it suppresses TGF‐β1 transcription and Smad3 activation while upregulating Smad7, ultimately reducing fibronectin deposition, and renal hypertrophy.

Rather than acting through isolated molecular targets, the renoprotective effects of NAR in DN appear to reflect an integrated modulation of a pathophysiological cascade. Sustained hyperglycemia initiates a self‐amplifying cycle of metabolic dysregulation, oxidative stress, inflammatory activation, and extracellular matrix accumulation that ultimately culminates in progressive renal fibrosis and functional decline (Efiong et al. 2025; Liu et al. 2025). NAR appears to intervene at multiple nodes within this network (Figure 8).

#### 4.1.1 NAR May Disrupt the Glucose‐Oxidative Pathway

This review found that NAR significantly reduced fasting blood glucose (SMD = −3.10). Glycemic control is widely recognized as one of the primary therapeutic targets in the management of DN. While NAR's effect on body weight was not statistically significant, its glucose‐lowering activity may attenuate glucotoxicity‐driven mitochondrial dysfunction and reactive oxygen species (ROS) generation in renal parenchymal cells, thereby reducing the oxidative burden that propagates downstream injury (Dwivedi and Sikarwar 2025). Experimental studies have shown that, in diabetic rats, NAR can restore pancreatic β‐cell mass and enhance glucose‐stimulated insulin secretion in pancreatic islets through activation of an estrogen receptor β (ERβ)‐dependent pathway, thereby improving glucose metabolism (Lin et al. 2023; Park et al. 2022). Other studies have reported that NAR significantly increases the phosphorylation of AMP‐activated protein kinase in skeletal muscle cells, promoting glucose uptake in peripheral tissues (Zygmunt et al. 2010). In addition, studies have demonstrated that NAR improves DN in lipid profiles, including reductions in total cholesterol, triglycerides, and low‐ and very‐low‐density lipoproteins, as well as increases in high‐density lipoproteins (Roy et al. 2016). By ameliorating systemic glucose and lipid metabolism, NAR attenuates the hyperglycemia‐driven activation of protein kinase C, advanced glycation end product formation, and polyol pathway flux, processing that are central to the initiation of renal oxidative stress in DN (Efiong et al. 2025). Therefore, NAR may regulate glucose homeostasis through coordinated effects on pancreatic function, insulin secretion, and glucose uptake in peripheral tissues, thereby interrupting the pathological reaction of the glucose‐oxidative pathway.

#### 4.1.2 NAR May Attenuate Oxidative Stress‐Driven Inflammatory and Fibrotic Signaling

Hyperglycemia‐induced excessive production of reactive species and the subsequent release of multiple inflammatory mediators constitute a critical component of DN pathogenesis (Kanwar et al. 2011; Li et al. 2023). In the integrated pathophysiology of DN, mitochondrial ROS overproduction triggered by glucotoxicity serves as a convergent signaling node that activates redox‐sensitive transcription factors, most notably nuclear factor kappa‐B (NF‐κB), which in turn drives the expression of pro‐inflammatory cytokines (tumor necrosis factor‐α, interleukin‐1β, interleukin‐6) and chemokines that recruit macrophages and promote tubulointerstitial inflammation (Liu et al. 2025; Yang et al. 2025). Simultaneously, ROS and inflammatory mediators activate latent TGF‐β1 and downstream Smad2/3 signaling, stimulating the synthesis of extracellular matrix proteins including fibronectin and collagen, ultimately leading to glomerulosclerosis and tubulointerstitial fibrosis (Qiu et al. 2023). This meta‐analysis demonstrated that NAR reduced renal MDA levels while increasing GSH content, suggesting that NAR effectively alleviates oxidative stress in the kidneys of DN models. These antioxidant effects are not merely parallel benefits; rather, they likely constitute a mechanistic prerequisite for NAR's anti‐inflammatory and antifibrotic actions, as ROS scavenging diminishes NF‐κB activation and TGF‐β1 signaling (Rispoli et al. 2025). In parallel, previous studies have reported reductions in pro‐inflammatory cytokines, including interleukin‐1β, tumor necrosis factor‐α, and interleukin‐6, in plasma and renal tissue following NAR treatment (Li et al. 2018; Ran et al. 2019). The antioxidative and anti‐inflammatory effects of NAR are also closely associated with its ability to inhibit NF‐κB p65 nuclear translocation and the subsequent expression of downstream inflammatory mediators (Tsai et al. 2012). Thus, NAR may appear to disrupt the oxidative stress‐inflammation‐fibrosis axis at its origin by quenching ROS, thereby preventing the feed‐forward loop that amplifies renal injury.

As for the indicators of SOD and CAT, which did not reach statistical significance in this meta‐analysis. It is speculated that NAR may exert its antioxidative effects primarily by directly scavenging reactive oxygen species and enhancing nonenzymatic antioxidant defenses (e.g., GSH regeneration) rather than by uniformly activating endogenous antioxidant enzyme systems across all experimental contexts. Besides, there are also methodological differences across studies, such as variations in renal tissue sampling sites (cortex vs. medulla), homogenization procedures, and enzyme activity assay techniques, which may contribute to inconsistent results.

#### 4.1.3 NAR May Suppress Renal Fibrosis via the TGF‐β/Smad Pathway

This meta‐analysis demonstrated that NAR significantly downregulated renal FN and TGF‐β1 expression, indicating a clear antifibrotic potential in DN. The observed reduction in KI further supports the beneficial role of NAR in attenuating renal hypertrophy and structural remodeling. Within the integrated pathophysiological framework, TGF‐β1 upregulation represents a terminal effector event that is tightly coupled to upstream metabolic and oxidative disturbances. Hyperglycemia and ROS stimulate latent TGF‐β1 activation, while inflammatory cytokines further amplify TGF‐β receptor signaling and Smad3 phosphorylation, leading to epithelial‐to‐mesenchymal transition and extracellular matrix accumulation (Meng, Tang, et al. 2015; Qiu et al. 2023). The TGF‐β/Smad signaling pathway is a central axis driving renal interstitial fibrosis in DN (Feng et al. 2024; He et al. 2024). Under diabetic conditions, TGF‐β/Smad3 signaling is markedly activated, accompanied by the loss of its inhibitory regulator Smad7, ultimately leading to pronounced glomerular and tubulointerstitial fibrosis (Chen et al. 2011; Goumenos et al. 2002). Previous studies have shown that NAR can significantly suppress Smad3 activation while upregulating Smad7 expression in DN renal tissue, suggesting that NAR acts as an effective Smad3 inhibitor (Shin and Shin 2024). In addition, NAR has been reported to downregulate TGF‐β1 gene transcription, interfere with TGF‐β1 binding to its receptors, and thereby inhibit the TGF‐β/Smad3 signaling cascade (Meng, Zhang, et al. 2015). By suppressing TGF‐β1 at both transcriptional and receptor levels, NAR not only reduces direct profibrotic signaling but also interrupts the cross talk between inflammatory and fibrotic pathways, given that TGF‐β1 itself can further stimulate ROS production and NF‐κB activity, creating a self‐perpetuating injury loop (Efiong et al. 2025). Through these mechanisms, NAR may reduce the synthesis of extracellular matrix components such as FN and slow the progression of glomerular and interstitial fibrosis.

### 4.2 Nonlinear Dose–Response Relationship of NAR Treatment in DN

Subgroup analyses revealed an atypical dose–response relationship of NAR in rodent models of DN. Specifically, the dose < 50 mg/kg exhibited superior regulatory effects on blood glucose and GSH compared with the dose of 50–100 mg/kg. In contrast, improvements in 24 h‐Upro, Scr, SOD, and KI were more pronounced in the 50–100 mg/kg dose group. Several explanations may try to understand this phenomenon. First, NAR may exhibit a saturation effect or a “therapeutic window,” whereby excessively high doses result in diminished efficacy due to poor solubility or reduced bioavailability. Second, different pathological processes may display varying dose sensitivities, with renal functional protection potentially requiring higher doses than metabolic or antioxidative modulation. Third, none of the included studies reported pharmacokinetic parameters, making it impossible to exclude confounding effects related to differences in systemic exposure or plasma drug concentrations.

### 4.3 Time‐Dependent Effects of NAR Treatment in DN

Based on this meta‐analysis, long‐term intervention (8–12 weeks) demonstrated superior effects across most outcomes, including blood glucose, 24 h‐Upro, Scr, BUN, and KI, which is consistent with the clinical understanding that reversing or attenuating the pathological changes of chronic kidney disease requires sustained therapeutic exposure. In contrast, short‐term intervention (4–8 weeks) produced a significant increase in renal GSH levels, suggesting that the antioxidative effects of NAR may manifest more rapidly. This time‐dependent pattern indicates that the selection of observation windows in preclinical studies should be aligned with specific study endpoints and also provides important methodological insights for the design of future clinical trials.

### 4.4 Limitations and Future Prospects

Although this meta‐analysis provides preliminary evidence supporting the multi‐target renoprotective effects of NAR in DN, several limitations within the evidence base warrant consideration. Firstly, significant heterogeneity was observed across outcome measures. Although subgroup analyses were conducted to investigate potential sources of variability, residual heterogeneity remained. This may be attributable to differences in animal strains, dosing regimens, and non‐standardized outcome assessment methods. Future studies should provide detailed reports on animal sex and age, standardize administration routes and treatment windows for both NAR and control groups, and clearly define assessment time points to improve comparability and facilitate data pooling.

Secondly, the methodological quality of the included studies was generally low to moderate. Many studies did not adequately report important design elements, such as randomization procedures, allocation concealment, or blinding, potentially undermining internal validity and elevating the risk of outcome assessment bias. Future research should prioritize prospective protocol registration, adhere strictly to registered protocols, and thoroughly report all methodological details to minimize bias and avoid selective outcome reporting.

Thirdly, the evidence presented here is entirely derived from rodent models of DN, which cannot fully recapitulate the complexity of human diabetic kidney disease. Rodent models predominantly rely on streptozotocin‐induced β‐cell injury or genetically engineered hyperglycemia, whereas human DN develops over decades in the context of heterogeneous genetic backgrounds, comorbidities (e.g., hypertension, dyslipidemia), and polypharmacy (Betz and Conway 2016). Species‐specific differences in drug metabolism, renal physiology, and immune responses further limit the direct translatability of preclinical efficacy to human populations (Hackam and Redelmeier 2006).

Fourthly, publication bias poses an underlying threat to the validity of our pooled estimates. Egger's test indicated an asymmetric funnel plot for blood glucose (*p* < 0.001), and the trim‐and‐fill method was used to evaluate the possible impact of publication bias. While the adjusted effect size appeared directionally consistent with the original estimate, this statistical correction does not eliminate the underlying risk of inflated effect sizes (Sena et al. 2010). Animal studies with small sample sizes and positive findings are more likely to be published, whereas null or negative results often remain unpublished, leading to an overrepresentation of beneficial outcomes (Korevaar et al. 2011). Furthermore, small study effects, wherein fewer animals tend to yield larger treatment effects, can substantially exaggerate apparent efficacy in preclinical research (Sterne et al. 2000). Given that the included studies predominantly employed small sample sizes (typically 5–10 per group) and none were prospectively registered, selective reporting and publication bias may have inflated our pooled SMD estimates. Consequently, the magnitude of NAR's renoprotective effects reported here likely represents an overestimation of the true biological effect.

Further, although NAR has been widely investigated for its glucose‐lowering, anti‐inflammatory, and antifibrotic properties, its dose–response relationship remains unclear, and pharmacokinetic data are largely insufficient. None of the included studies measured plasma or tissue NAR concentrations, precluding the establishment of exposure‐response relationships and raising concerns about whether rodent doses achieve clinically relevant systemic exposure in humans. Future studies should integrate DN animal modeling with pharmacokinetic assessments to establish dose–response relationships and pharmacokinetic profiles that may better inform clinical translation.

Finally, the existing evidence on NAR treating DN is restricted to the preclinical phase. Given the substantial methodological limitations, small sample sizes, and the risk of publication bias identified in this review, any inference regarding NAR's clinical utility in DN remains hypothetical and premature. Future efforts should focus on rigorous preclinical validation before advancing to clinical research. Should preclinical findings prove robust in larger, multispecies, and prospectively registered studies, well‐designed early‐phase clinical trials could subsequently evaluate NAR's pharmacokinetics, safety, and preliminary efficacy in patients with diabetic kidney disease. However, at present, NAR cannot be recommended as an adjunct or alternative to standard of care therapies for DN based on the available rodent evidence alone.

## 5 Conclusion

This meta‐analysis, which included 12 studies, demonstrates that NAR exerts significant renoprotective effects in rodent models of DN. Specifically, NAR effectively reduces blood glucose, improves renal function, and alleviates renal oxidative stress and fibrosis. Therapeutic benefits were more pronounced at doses of 50–100 mg/kg and with intervention durations of 8–12 weeks. However, these findings are derived from a limited evidence base characterized by small sample sizes, substantial heterogeneity, generally low‐to‐moderate methodological quality, and a high probability of publication bias. While NAR shows multi‐target therapeutic potential in preclinical models, its efficacy and safety in human diabetic kidney disease remain entirely unproven. Therefore, NAR should be regarded as a candidate for further preclinical and early‐phase clinical investigation, not as an established therapeutic intervention for DN.

## Author Contributions

**Ling Yue:** investigation, formal analysis, writing – original draft. **Yuzhu Qu:** conceptualization, funding acquisition, writing – original draft, writing – review and editing, project administration. **Kongmei Ou:** investigation. **Yiting Wang:** methodology. **Benxiang He:** conceptualization, writing – review and editing. **Jie Chen:** writing – original draft, formal analysis. **Xinyue Zhang:** investigation. **Runjia He:** methodology.

## Funding

This study was supported by the Sichuan Science and Technology Program (No. 2026NSFSC1859), China Postdoctoral Science Foundation (No. 2025MD774039), and Joint Innovation Fund of Health Commission of Chengdu and Chengdu University of Traditional Chinese Medicine (No. WXLH202405009 and No. WXLH202402013).

## Disclosure

Author approval and responsibility statement: All authors have read and approved the final version of the manuscript. Corresponding Author had full access to all of the data in this study and takes complete responsibility for the integrity of the data and the accuracy of the data analysis.

## Ethics Statement

The authors have nothing to report. This systematic review and meta‐analysis synthesized data exclusively from previously published rodent studies. No human subjects, human biological materials, or live animals were directly involved in this research. All original animal studies were conducted in accordance with institutional guidelines for the care and use of laboratory animals, as reported by the respective primary study authors.

## Conflicts of Interest

The authors declare no conflicts of interest.

## Data Availability

The data that support the findings of this study are available from the corresponding author, upon reasonable request.
